# One-Shot Fabrication of Polymeric Hollow Microneedles by Standard Photolithography

**DOI:** 10.3390/polym13040520

**Published:** 2021-02-09

**Authors:** Principia Dardano, Selene De Martino, Mario Battisti, Bruno Miranda, Ilaria Rea, Luca De Stefano

**Affiliations:** 1 Institute of Applied Sciences and Intelligent Systems, Italian National Council of Research, 80131 Naples, Italy; Bruno.miranda@na.isasi.cnr.it (B.M.); ilaria.rea@na.isasi.cnr.it (I.R.); luca.destefano@na.isasi.cnr.it (L.D.S.); 2 Materias S. R. L., 80100 Naples, Italy; Selene.DeMartino@Materias.it (S.D.M.); Mario.Battisti@Materias.it (M.B.); 3 Department of Electrical Engineering and Information Technology, Università degli Studi di Napoli “Federico II”, 80100 Naples, Italy

**Keywords:** microneedles, microfabrication, photolithography, optical simulation

## Abstract

Microneedles (MNs) are an emerging technology in pharmaceutics and biomedicine, and are ready to be commercialized in the world market. However, solid microneedles only allow small doses and time-limited administration rates. Moreover, some well-known and already approved drugs need to be re-formulated when supplied by MNs. Instead, hollow microneedles (HMNs) allow for rapid, painless self-administrable microinjection of drugs in their standard formulation. Furthermore, body fluids can be easily extracted for analysis by a reverse use of HMNs, thus making them perfect for sensing issues and theranostics applications. The fabrication of HMNs usually requires several many-step processes, increasing the costs and consequently decreasing the commercial interest. Photolithography is a well-known fabrication technique in microelectronics and microfluidics that fabricates MNs. In this paper, authors show a proof of concept of a patented, easy and one-shot fabrication of two kinds of HMNs: (1) Symmetric HMNs with a “volcano” shape, made by using a photolithographic mask with an array of transparent symmetric rings; and (2) asymmetric HMNs with an oblique aperture, like standard hypodermic steel needles, made by using an array of transparent asymmetric rings, defined by two circles, which centers are slightly mismatched. Simulation of light propagation, fabrication process, and preliminary results on ink microinjection are presented.

## 1. Introduction

The transdermal drug delivery way has the main advantage of delivering drugs into the systemic circulation through the skin—avoiding the potential degradation associated with the gastrointestinal tract and the first-pass liver effects. Moreover, for patients, the transdermal way has many advantages, such as painlessness, low invasiveness, and easy self-administration [1,2,3,4]. However, the stratum corneum is the first skin barrier, which only small and lipophilic molecules can overcome [5,6]. On the other hand, the analysis of epidermal interstitial fluids (ISF) was demonstrated significant and comparable to both plasma and serum analysis for sensing purposes [7]. A recent proteomic characterization of the epidermal ISF has revealed the presence of 407 proteins, which less than one percent have been identified only into the ISF, confirming that the ISF is strictly connected to human blood [8].

Microneedles (MNs) are needles at the microscale, which height is enough to overcome the stratum corneum and reach the ISF, but not the nerves. MNs based devices have been proposed to interact with the human body through the skin for both drug administration and ISF analysis. This kind of painless door to the human body gives minimal skin trauma, has a low probability of pathogens introduction, and it is easily disposable [9,10,11]. Nowadays, the MNs are an emerging technology in pharmaceutics and biomedicine, quite ready for global commercialization. Currently, a lot of fabrication methods have been used to realize swelling, solid, coated, and dissolvable MNs, such as etching, micromolding, thermal drawing, laser lithography, centrifugal drawing, 3D printing, and so on [12,13,14,15,16,17,18,19]. However, swelling, soluble, and coated solid microneedles utilization in drug delivery often needs a new formulation of the drugs even if well-known and already approved. Moreover, these kinds of MNs also suffer from the low administrable dose and rate of administration.

Unlike solid MNs, which can have at most only porous structures, hollow MNs (HMNs) have an inner cavity, i.e., a whole hollow core. Sizes of the inner cavities strongly depend on the material and fabrication method, and the inner diameter varies from 30% to 70% of the outer diameter, generally from 60 μm to 300 μm [20,21,22,23]. Customized inner cavities open the road to deliver bigger molecules, such as macromolecules or nanoparticles, in the fastest possible way, with a proper flow [20,21,22,23,24,25]. HMNs allows painless and self-administrable microinjection of drugs in their standard formulation. On the other hand, the extraction, analysis, and interaction with body fluids using HMNs make them perfect for sensing issues and theranostics applications [8,20,21,22,23,24,25]. Fabrication of HMNs usually needs many-step processes, thus increasing the overall costs and consequently decreasing the potential commercial interest. Photolithography is a well-known fabrication technique in microelectronics and microfluidics, that enables the fabrication of solid MNs of several shapes and designed lengths. In [26,27,28], authors proposed a very easy fabrication method for solid and swelling MNs based on standard photolithography, where a mixture of Polyethylene glycol diacrylate (PEGDA) and a commercial photoinducer has been used as a negative photoresist, without any etching step. PEGDA is a biocompatible polymer that solidifies at room temperature in the presence of a photo-initiator using exposure to ultraviolet (UV) light for a few seconds.

In this paper, we show a proof of concept of a patented, easy and one-shot fabrication of PEGDA based HMNs. In this case, any etching step is avoided, and the inner cavity of the HMNs is fabricated at the same time as the external structure of the needles. Two kinds of HMNs are presented: The symmetric HMNs (SHMNs) with a “volcano” shape were made by using a mask by using an array of transparent symmetric rings; and the asymmetric HMNs (AHMNs) with an oblique aperture, just like the standard hypodermic steel needles, were made by using an array of transparent asymmetric rings, defined by two circles, which centers are slightly mismatched. We demonstrate by numerical simulation of UV light propagation the role of the photolithographic mask in a simple fabrication process. Moreover, mechanical properties and preliminary results on ink microinjection are presented.

## 2. Materials and Methods

### 2.1. Materials

All chemicals are commercially available and used as received. We purchased PEGDA with number average molecular weight, Mn = 250, Bovine gelatin type B, and P7793-PARAFILM^®^ M by Merck KGaA, Darmstadt, Germany. 2-Hydroxy-2-methyl-1-phenyl-propan-1-one (DAROCUR© 2021) has been purchased by BASF SE, Ludwigshafen am Rhein, Germany. The DAROCUR© is a liquid photo-initiator that is used with PEGDA at 2% (*v/v*) to fabricate HMNs and their substrate. The use of DAROCUR© to initiate the photo-polymerization of the pre-polymer solution has been largely documented [29,30].

### 2.2. Direct Photolithographic Methods

In this work, we proposed a simple method based on direct photolithography to realize HMNs in a one-step fabrication process. As previously described in detail [28], the mixture of PEGDA and DAROCUR© has been used as a negative photoresist, obtaining the needles with their inner cavities, due to the mask geometry, without any etching treatment.

**Figure 1:**
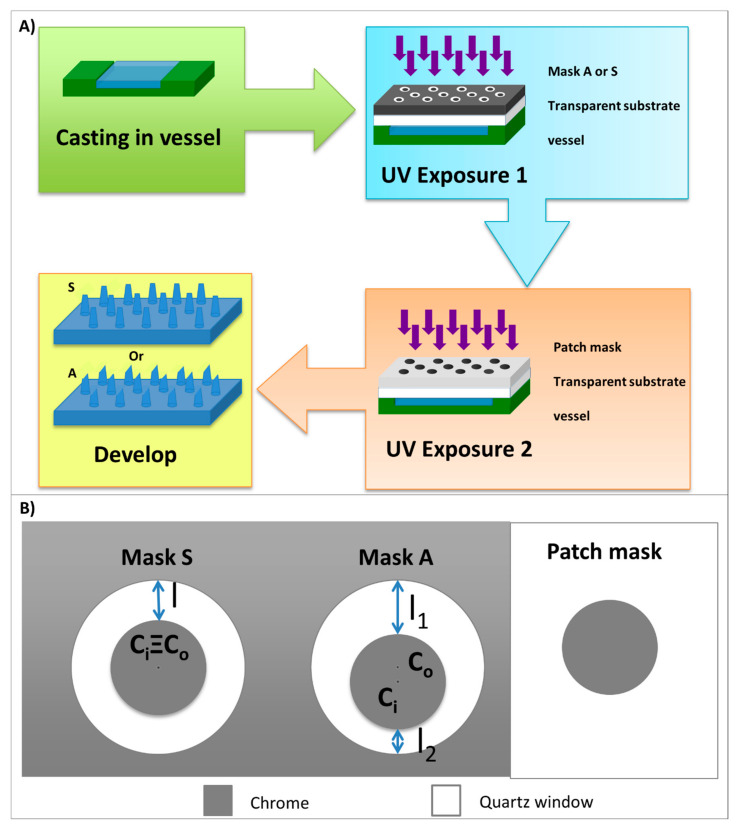
(**A**) Flow charts of the device fabrication: The pre-polymer solution, initially in a liquid state, was cast into a vessel and hardened by two UV exposure. First, UV exposure defines hollow microneedles (HMNs), the second one defines the pierced patch substrate. Finally, samples were developed in deionized water and dried with nitrogen; (**B**) two kinds of photolithographic masks have been used: Mask S was an array of microholes with symmetric ring shape; in this case, the ring was defined by two circles, whose centers were perfectly aligned. Mask A was an array of microholes with asymmetric ring shape; two circles, whose centers were slightly mismatched, defined the ring. The patch mask allowed the realization of pierced patch substrate. C_i_ is the center of the inner circle, C_o_ is the center of the outer circle, l is the width of the symmetric aperture in Mask S, l_1_ and l_2_ are the maximum and the minimum width of the asymmetric aperture in Mask A.

Figure 1A reports the flow charts of the device fabrication. The pre-polymer solution, initially in a liquid state, was cast into a vessel and hardened by UV exposure with MA6/BA6 mask aligner (by Karl Suss, SUSS MicroTec SE, Garching, Germany) at 365 nm. The vessel has a dual function, i.e., it is both a container for the liquid pre-polymer solution and a spacer useful to grow the needles’ height. It should be noted that a simple UV lamp could be used to realize the presented HMNs, and generally, the process does not require the use of a clean room, but the alignment of the drilled substrate with HMNs requires a mask alignment system and a biomedical device must always be manufactured in a safe and controlled environment.

Two kinds of photolithographic masks have been used to fabricate different HMNs. Symmetric HMNs, with a “volcano” shape, were made by using an array of transparent symmetric rings: In Mask S, the outer and inner diameters of circles defining the symmetric ring were 500 μm and 380 μm, respectively, and the centers were perfectly aligned (Figure 1B). Then, in this case, the ring aperture width was 60 μm.

Asymmetric HMNs, with an oblique aperture, were made by using an array of transparent asymmetric rings: In Mask A, the ring was defined by two circles, which centers were slightly mismatched. The outer and inner diameters of circles are 825 μm and 600 μm, respectively, and the ring aperture width varied from 75 μm to 150 μm (Figure 1B). In Figure 1B, C_i_ is the center of the inner circle, C_o_ is the center of the outer circle, l is the width of the symmetric aperture in Mask S, l_1_ and l_2_ are the maximum and the minimum width of the asymmetric aperture in Mask A.

A second exposure has been performed to fabricate a pierced patch as a substrate for MNs (Figure 1B).

The exposures were made by a mask-aligner in soft contact or close proximity to prevent contamination of the mask. Finally, samples were developed in deionized water and dried with nitrogen.

The dependence of HMNs, symmetrical and asymmetrical, height and shape on the dose of radiation has been studied at a power density of the UV source fixed at 17 mW/cm^2^ by changing the exposure time: 7, 9, 13, and 18 s, for SHMNs and 3, 5, 9, and 18 s, for AHMNs.

### 2.3. Optical Simulations

The considered optical systems were constituted by a microaperture array of the photolithographic mask (schematized as a chromium layer), a UV transparent substrate (glass coverslip), solid and liquid PEGDA. The UV light propagation through the optical systems was simulated by using the optics module of COMSOL Multiphysics^®^ software (by COMSOL, Inc., Burlington, USA). UV light was modeled as a plane wave with propagating wave vector parallel to the height axis of the MN, defined as x axis. The plane wave wavelength was set at λ_0_ = 365 nm, and the maximum size of the mesh elements was fixed at λ_0_/5. The solid PEGDA refractive index was set at 1.5 (as measured by spectroscopic ellipsometry, data not shown here), while the liquid PEGDA refractive index was set at 1.46, as reported in the material datasheet. The refractive index of the glass coverslip, used as transparent support, was set at 1.5. The aperture section, l, was set at 60 μm for SHMNs. Meanwhile, for AHMNs, the aperture sections were set at 150 μm and 75 μm for the major aperture, l_1_, and minor aperture, l_2_, respectively (as reported in the masks in Figure 1. Unlike from SHMNs, in the case of AHMNs, two different aperture sections result from the radial section of the asymmetric ring apertures. Then, we simulated together with the two different apertures, corresponding to l_1_ and l_2_, to emphasize that different heights of the HMNs wall, corresponding to h_A1_ and h_A2_, can be simultaneously fabricated, and AHMN can be fabricated in a one-shot photolithographic step. In both cases, three scenarios were simulated: The first one relates to the not yet polymerized MN wall section, the second one considers a partially formed MN wall section, and the third one considers a fully formed MN wall section.

### 2.4. Characterization

#### 2.4.1. Image Analysis

Image of HMNs, their imprint in the indentation samples, and the ink into injection samples have been performed and analyzed by DM6 Optical Microscope system equipped by Leica DFC 7000T digital camera (by Leica, Wetzlar, Germany).

#### 2.4.2. Scanning Electron Microscopy

Micro and nano features of MNs have been evaluated from images by scanning electron microscopy (SEM). SEM images were performed at 5 kV accelerating voltage and 30 μm wide aperture by a field emission scanning electron microscope (by Carl Zeiss NTS GmbH 1500 Raith FESEM, Dortmund, Germany). Secondary emission detector has been used. The polymeric MNs were gold-coated to improve the conductivity, and thus, the definition of the SEM images. Some MN has been detached by the patch and imaged horizontally.

#### 2.4.3. Indentation and Injection Proof

Indentation tests have been performed on 8 folded Parafilm^®^ layers (by Merck KGaA, Darmstadt, Germany), which have been used as artificial skin as reported in [31] where mechanical properties of Parafilm^®^ have been proved to be very close to that of pigskin. Images of the first layers have been captured by an optical microscope.

Injection experiments have been performed on skin mimic gelatin samples, as done in [32]: Bovine gelatin type B (by Sigma Aldrich) was used to prepare hydrogel samples. Gelatin powder (30% weight/volume) was dissolved in a pH 7 Phosphate-Buffered Saline (PBS) solution with continuous stirring at 70 °C. The solution was then poured into cylindrical molds and kept at room temperature for 1 h to form the gel. HMNs fixed on a syringe-type system containing 1 M concentrated solution of Methylene Blue (MB) have been used for the indentation test. The system was manually pressed onto the gelatin mold, and the MB solution was injected into the hydrogel. The HMNs were removed from the gelatin mold, and the diffusion of MB solution into the hydrogel was analyzed by imaging gelatin samples and his transversal sections by DM6 Optical Microscope system (by Leica, Wetzlar, Germany).

## 3. Results

### 3.1. Optical Simulations

#### 3.1.1. SHMNs

**Figure 2:**
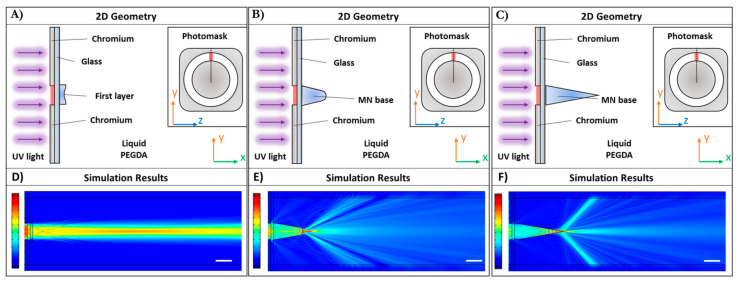
Symmetric HMNs (SHMNs) optical simulations: Schematization of the 2D geometries used to perform the simulations for the not yet polymerized (**A**), partially polymerized (**B**), and fully polymerized (**C**) SHMN wall section, respectively. The geometry considers only one aperture section of the photomask; thus, the aperture through which UV light propagates is 60 μm. Liquid PEGDA refractive index is 1.46, glass coverslip refractive index is 1.5, and the MN wall sections refractive index was varied from 1.5 at the center of the aperture, to 1.46 at the extremities. The simulation results show the spatial distribution of the light intensity for the not yet polymerized (**D**), partially polymerized (**E**), and fully polymerized MN wall section (**F**), respectively. The color bars represent the normalized intensities with respect to the incident field: The minimum value and maximum values are depicted in blue and red, respectively. The white scale bar is 100 μm.

In the case of SHMNs, a single aperture section, l, was considered into simulated 2D optical systems. Figure 2 resumes the 2D optical systems (Figure 2A–C) and simulation results (Figure 2D–F) for SHMNs. Considering that the UV-light propagation and polymerization were two phenomena that occurred in two extremely different timescales, the simulation of at least three scenarios was necessary to understand the polymerization working conditions and the focusing of the UV-light during the formation of the MNs. The mapping of the MNs refractive index allowed a better description and comprehension of the phenomenon. For that reason, as described in Section 2.1, three optical systems were simulated, and every simulation was run at a steady state.

In the first system (Figure 2A), a thin layer (10 μm) with refractive index, *n*, varying from a solid state (*n* = 1.5 at y = 0 μm) to a liquid state (*n* = 1.46 at y = ±30 μm) was considered. The simulation results show the spatial distribution of the light intensity (normalized respect to the incident intensity), where the minimum value and maximum values are depicted in blue and red, respectively. The white scale bar is 100 μm. It is evident in Figure 2D a pseudo-triangular shape in the light intensity distribution. Considering that the pre-polymer solution with the same UV illumination polymerized simultaneously with the same probability, the MNs wall section corresponding to the aperture section l showed a pseudo-triangular shape, as well as the light distribution (Figure 2D).

In the second system, a partial MNs wall section (schematized with the polygon reported in Figure 2B, with refractive index varying from *n* = 1.5 at y = 0 μm to *n* = 1.46 at y = ±30 μm, and height selected according to the first simulation (100 μm), was considered. In this case, simulations showed a light focalization slightly out of the partial MNs wall section that strongly triggers the PEGDA polymerization strictly over the tip, resulting in a triangular MNs wall section (Figure 2E). Finally, the entire MNs wall section (schematized as a triangle, 200 μm high, in Figure 2C was considered, and also, in this case, the refractive index varies from *n* = 1.5 at y = 0 μm to *n* = 1.46 at y = ±30 μm. In this last case, simulations showed a light focalization on the tip of MNs wall section that enables PEGDA polymerization in height (Figure 2F).

#### 3.1.2. AHMNs

**Figure 3:**
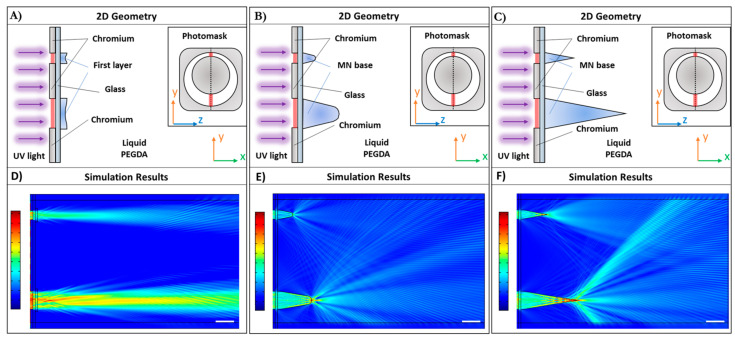
Asymmetric HMNs (AHMNs) optical simulation: Schematization of the 2D geometries used to perform the simulations for the not yet polymerized (**A**), partially polymerized (**B**), and fully polymerized (**C**) AHMN cross-section, respectively. The geometry considers two aperture sections l_1_ and l_2_ of the photomask, both laying on the same y axis, thus the apertures through which UV light propagates are 75 μm and 150 μm, respectively. The refractive index varies from 1.5 in the middle of the aperture sections to 1.46 at the extremities. The simulation results show the spatial distribution of the light intensity for the not yet polymerized (**D**), partially polymerized (**E**), and fully polymerized MN cross-section (**F**), respectively. The color bars represent the normalized intensities with respect to the incident field: The minimum value and maximum values are depicted in blue and red, respectively. The white scale bar is 150 μm.

In the case of AHMNs, two apertures, l_1_ and l_2_, were considered into the simulated 2D optical systems, both laying on the same y axis, and were set at 150 μm and 75 μm (Figure 2) for the major and minor aperture section, respectively. Analogously to SHMN case, three different optical systems were considered for AHMN: A first system (Figure 3A), with two thin layers (about 10 μm) corresponding the two apertures, with refractive index, *n*, varying from a solid state (*n* = 1.5 at the center of the apertures) to a liquid state (*n* = 1.46 at the border of apertures); a second system, with two partial MN wall sections, corresponding to the main significant MN cross-section, with refractive index varying from *n* = 1.5 to *n* = 1.46, and heights 125 μm and 250 μm, respectively (Figure 3B); and finally, two entire MN wall sections with height 250 μm and 500 μm, respectively, were considered (Figure 3C). In the case of AHMN, simulations showed different light intensity distribution corresponding to the two aperture sections, confirming that the height of the MN wall section depended on the aperture section width (Figure 3E,F). Then, in the AHMN, the asymmetric upper aperture of the needle was generated by the asymmetry into the ring aperture.

### 3.2. Device Fabrication Results

**Figure 4:**
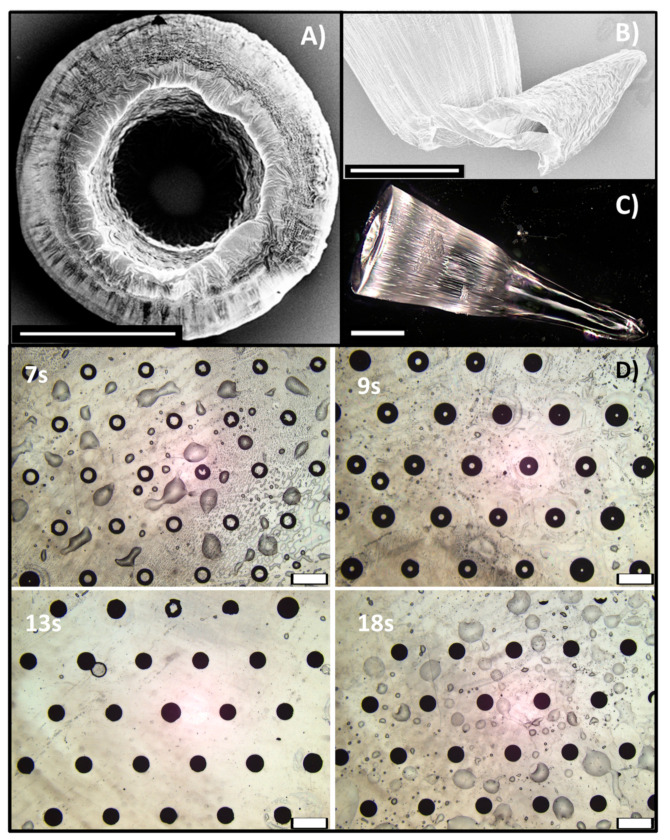
(**A**) SEM image of the top view of an SHMN exposed for 9 s: Volcano shape is well recognizable; (**B**) SEM image of the lateral view of a broken fully-formed SHMN (13 s), with closed aperture: The inner channel is present till the top of the MN; (**C**) optical image of a fully-formed SHMN (18 s): Transparence of PEGDA allow to see the inner channel; the scale bar is 250 μm in (**A**–**C**). (**D**) Optical image of the top view of SHMNs arrays at 7, 9, 13, and 18 s exposure time. The scale bar is 1000 μm.

**Figure 5:**
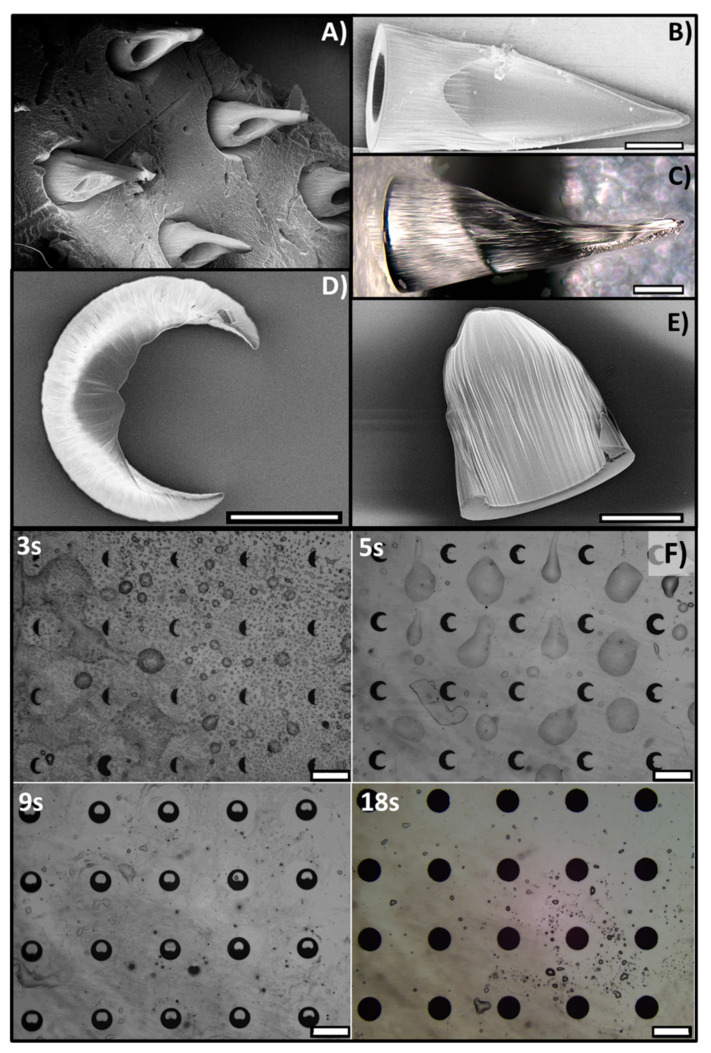
(**A**) SEM image of a tilted view of an AHMN exposed for 11.5 s with his patch: (**B**) SEM image of the lateral view of AHMN (9 s); (**C**) optical image lateral view of AHMN (11.5 s); (**D**,**E**) SEM image of the top view (**D**) and lateral view (**E**) of an AHMN exposed for 5 s; the scale bar is 250 μm. (**F**) Optical images of the top view of AHMNs arrays at 3, 5, 9, and 18 s exposure times: The dependence of the oblique upper aperture width by the exposure time is well recognizable. The scale bar is 1000 μm.

Main fabrication results are summarized in Figure 4 and Figure 5, for SHMNs and AHMNs, respectively, and in Table 1, where h_S_ and h_A_ are the MNs height obtained from mask type S and A, respectively.

#### 3.2.1. SHMNs

Mask S (symmetric rings in Figure 1B) enabled SHMNs array, which height and upper aperture depended on exposure time. For exposure time lower than 7 s, no MNs array appears; at 7 s of exposure time, only a low (100 μm high) volcano-shaped MNs with a wide upper aperture appears; for SHMNs exposed for 9 s, the volcano shape is well recognizable in some MNs even if the height is extremely irregular and vary between 150–240 μm (Figure 4A); for 13 and 18 s exposure time, fully-formed SHMN array appears with closed aperture and heights 1000 μm and 1500 μm, respectively (Figure 4B,C). However, in the optical images fully-formed SHMNs, the transparence of PEGDA allows us to see an inner channel (Figure 4C), and the SEM image of the lateral view of a broken MN in Figure 4B shows that the inner channel is present till the tip of the MN. Finally, in Figure 4D, optical images of a top view of SHMNs arrays at 7, 9, 13, and 18 s exposure time are presented and clearly show the dependence of the upper aperture width by the exposure time.

#### 3.2.2. AHMNs

Mask type A (asymmetric rings in Figure 1B) enabled in one only exposure HMNs with a lateral oblique aperture like in hypodermic syringes (Figure 5). Also, in this case, heights and lateral apertures were smaller as the exposure time increased. For exposure time lower than 9 s, no MNs array appears, although partially formed MNs stand up in correspondence of the wider aperture l_1_ of Mask A (see Figure 1B) for 3 s and 5 s exposure times (Figure 5D,E). For 9 s exposure time an HMNs array with a lateral oblique aperture is well recognizable from Figure 5A–C; the lateral oblique aperture has a shape like in hypodermic syringes resulting from the different aperture widths of Mask S section of the rings at 9 s exposure time: As well the aperture sections continuously range from l_1_ to l_2_, as well the heights continuously range from 1360 μm, h_A1_, to 240 μm, h_A2_. For 18 s exposure time, fully-formed AHMN array appears with closed apertures and tip height 1780 μm. Finally, in Figure 5F, optical images of the top view of AHMNs arrays at 3 s, 5 s, 9 s, and 18 s exposure times are presented and clearly show the dependence of the oblique upper aperture width by the exposure time.

### 3.3. Indentation and Injection Proof

**Figure 6:**
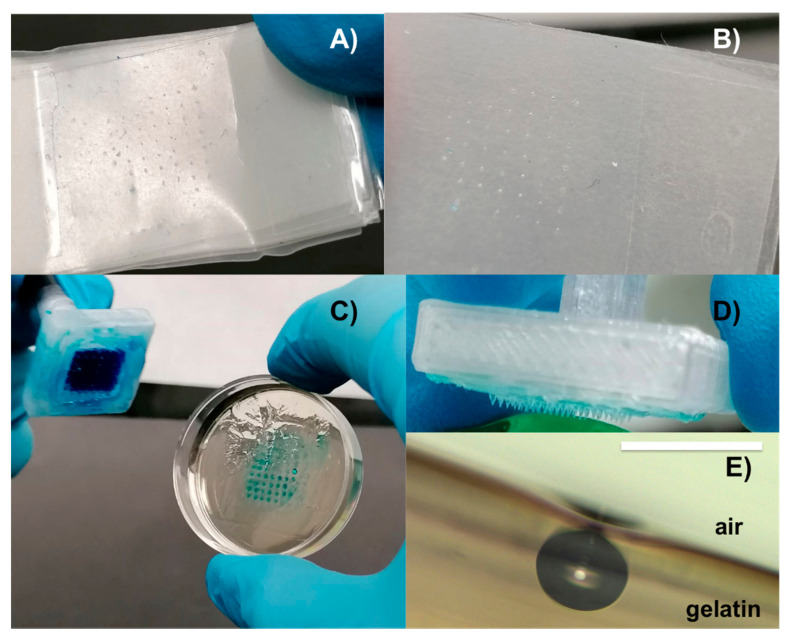
Indentation and injection tests: Photography of first (**A**) and fourth (**B**) layer of Parafilm^®^: the indention print is well visible in both cases; photography of the injection test: The ink jet is visible into the gelatin sample (**C**); photography of a AHMNs device after the injection test (**D**); the optical image of a cross-section of the gelatin sample with an ink bubble (**E**). The scale bar is 250 μm.

Indentation test performed with MN devices on Parafilm^®^ folded layers revealed an MNs print till the fourth layer, i.e., penetration of about 600 μm. In Figure 6A,B are reported photography of the first and fourth layer of Parafilm^®^, respectively.

Moreover, Figure 6C–E, shows images captured after MNs penetration in gelatin and the ink microinjections. MNs efficiently penetrate into the gelatin samples and can release the blue dye. In fact, the photo of the gelatin sample, in Figure 6C, shows the ink jet print of the whole device. Moreover, a cross-section of the gelatin sample with an ink bubble from a single MN is shown in the optical image, in Figure 6E. Finally, in Figure 6D, no damage is visible on the MNs device after the injection test.

## 4. Discussion

A mixture of PEGDA and a commercial photo-initiator as an ordinary photoresist has been used to fabricate suitable MNs, without any etching step. In fact, PEGDA is a biocompatible polymer that solidifies in the presence of a photo-initiator when exposed for a few seconds to UV light at room temperature. The photolithographic method resulted in a particularly flexible process to realize HMNs based devices. In fact, the same liquid photosensitive polymeric solution was used both to fabricate HMNs and their patch support, opening the road to a wide range of integrated microfluidics devices. SHMNs, with a “volcano” shape, and AHMNs, with an oblique aperture, are both fabricated by using standard photolithographic masks with transparent rings. Only the alignment or the mismatch of the centers of the circles defining the transparent rings during the UV exposure resulted in different shapes of HMNs, as proved by the optical simulation and fabrication results. MNs heights and aperture widths were closely connected with the dose exposure, which enabled the possibility to tune the flow rate of microinjections simply by using a suitable exposure time, as well as to encapsulate liquid formulation in the full closed HMNs obtained with high exposure time.

## 5. Conclusions

In this paper, we showed the proof of concept of a patented, easy, and one-shot photolithographic fabrication of two kinds of PEGDA based HMNs: Symmetric HMNs (SHMNs), with a “volcano” shape, and asymmetric HMNs (AHMNs), with an oblique aperture. Using standard photolithographic masks with an array of transparent rings, defined by two circles, which centers are perfectly aligned or slightly mismatched, fabrication of SHMNs or AHMNs, respectively, were enabled. MNs heights and aperture widths were closely connected with the dose exposure. In fact, lower exposure times (9 s) at 17 mW/cm^2^ resulted in wider upper aperture (till the not perfect formation for AHMNs in the extreme case of very low exposure time), and then, the possibility to tune the flow rate of microinjections.

High exposure time (18 s) at 17 mW/cm^2^ completely closes the upper aperture, in both symmetric and asymmetric cases, even if an inner cavity was ever found. In this case, the possibility of encapsulating liquid formulation in it is enabled. Finally, penetration and injection tests revealed the efficiency of penetration into skin mimic gelatin samples, as well as the diffusion of blue dye. Moreover, extracted HMNs by skin mimic samples proved not damaged during the test.

## 6. Patents

Luca De Stefano, Principia Dardano, Luigi Nicolais, Hollow microneedle for transdermal delivery of active molecules and/or for the sampling of biological fluids and manufacturing method of such hollow microneedle, World Patent WO2019243915A1, 2018.

## Figures and Tables

**Table 1:** Resulting MNs Heights vs. exposure time for Mask S and Mask A.

Time (s)	Height (μm)		
	h_S_^1^	h_A1_^2^	h_A2_^2^
3		160	0
5		610	0
7	100		
9	150–240	1360	240
13	1000		
18	1500	1780	1780

^1^ Height h_S_ correspond to l aperture in Mask S; ^2^ Height h_A1_ and h_A2_ correspond to l_1_ and l_2_ apertures in Mask A, see Figure 1B.

## Data Availability

Data is contained within the article.
